# Reduction in Synovitis Following Genicular Artery Embolization in Knee Osteoarthritis: A Prospective Ultrasound and MRI Study

**DOI:** 10.3390/diagnostics14222564

**Published:** 2024-11-15

**Authors:** Louise Hindsø, Per Hölmich, Michael M. Petersen, Jack J. Xu, Søren Heerwagen, Michael B. Nielsen, Robert G. C. Riis, Adam E. Hansen, Lene Terslev, Mikkel Taudorf, Lars Lönn

**Affiliations:** 1 Department of Radiology, Rigshospitalet, Blegdamsvej 9, 2100 Copenhagen, Denmark; soeren.heerwagen@regionh.dk (S.H.); mbn@dadlnet.dk (M.B.N.); robert.gabriel.coumine.riis.02@regionh.dk (R.G.C.R.); adam.espe.hansen@regionh.dk (A.E.H.); mikkel.taudorf@regionh.dk (M.T.); lars.birger.loenn@regionh.dk (L.L.); 2 Faculty of Health and Medical Sciences, University of Copenhagen, Blegdamsvej 3B, 2100 Copenhagen, Denmark; per.hoelmich@regionh.dk (P.H.); michael.moerk.petersen@regionh.dk (M.M.P.);; 3 Sports Orthopedic Research Center—Copenhagen, Department of Orthopedic Surgery, Copenhagen University Hospital, Amager & Hvidovre Hospital, Kettegård Alle 30, 2650 Hvidovre, Denmark; 4 Department of Orthopedic Surgery, Rigshospitalet, Blegdamsvej 9, 2100 Copenhagen, Denmark; 5 Department of Radiology, Zealand University Hospital—Slagelse, Fælledvej 11, 4200 Slagelse, Denmark; jjx@regsj.dk; 6 Center for Rheumatology and Spine Diseases, Rigshospitalet, Valdemar Hansens Vej 17, 2600 Glostrup, Denmark

**Keywords:** trans-arterial embolization, inflammation, knee osteoarthritis, magnetic resonance imaging, ultrasound

## Abstract

Background/Objectives: Genicular artery embolization (GAE) has demonstrated potential as a treatment for knee osteoarthritis by targeting inflammation and pain, although current evidence remains limited. This study used imaging biomarkers to objectively assess synovitis and possible ischemic complications following GAE. Methods: This was a prospective, single-center trial including participants with mild-to-moderate knee osteoarthritis. Ultrasound, contrast-enhanced (CE), and non-CE-MRI were performed two days before and one and six months after GAE. Ultrasound biomarkers included synovial hypertrophy, effusion, and Doppler activity. A combined effusion-synovitis score was assessed on non-CE-MRI, while CE-MRI allowed differentiation between synovium and effusion and was used to calculate whole-joint and local synovitis scores. The post-GAE MRIs were reviewed for ischemic complications. Results: Seventeen participants (aged 43–71) were treated. Significant reductions were observed in ultrasound-assessed synovial hypertrophy and Doppler activity, as well as in CE-MRI local and whole-joint synovitis scores. While reductions in effusion were noted in both ultrasound and MRI, these changes did not reach statistical significance. At one month, MRI revealed three cases of nonspecific osteonecrosis-like areas, which resolved completely by six months. Conclusions: This study demonstrated a reduction in synovitis and no permanent ischemic complication following GAE in knee osteoarthritis. Larger studies with longer follow-up are needed to confirm the long-term efficacy and safety of the procedure.

## 1. Introduction

Knee osteoarthritis (OA) is a degenerative joint disease affecting millions of individuals worldwide, with its incidence anticipated to rise, thereby increasing the socioeconomic burden on healthcare systems [1,2,3,4]. Symptoms are characterized by pain, stiffness, limited joint mobility, and muscle weakness [5,6]. Key risk factors include advanced age, female sex, obesity, previous knee injuries, and occupational or sports-related activities [2,5]. Once understood as a simple wear-and-tear condition, knee OA is now recognized as a complex disease influenced by inflammatory and molecular factors. [7,8]. Diagnosis typically involves physical examination and X-ray, while advanced imaging techniques such as magnetic resonance imaging (MRI), ultrasound (US), and computed tomography (CT) may be utilized in complex cases [9,10].

Current treatment options for knee OA include non-pharmacological strategies (e.g., exercise, weight loss, self-management), pharmacological interventions (e.g., oral pain relievers, anti-inflammatory medication), intra-articular injections (e.g., corticosteroids, hyaluronic acid), and surgical options for more advanced cases [3,5,6]. However, many patients do not experience sufficient relief with these treatments, highlighting the need for more effective therapeutic options.

Genicular artery embolization (GAE) is a minimally invasive procedure targeting neovessels associated with chronic inflammation. Preliminary findings indicate that GAE may reduce pain and improve function in patients with knee OA [11,12,13]. While the exact mechanisms are still under investigation, a reduction in synovitis is believed to play a significant role [12,13,14,15]. Most research has focused on patient-reported outcomes; however, incorporating imaging biomarkers of synovitis may provide an additional and objective assessment of GAE efficacy [12,16].

Two studies have demonstrated reductions in synovitis after GAE using the semi-quantitative scoring system WORMS (Whole-Organ Magnetic Resonance Imaging Scores [17]), which evaluates effusion and synovial thickening on non-contrast-enhanced MRI (non-CE-MRI) [13,18]. However, contrast-enhanced MRI (CE-MRI) offers a more detailed assessment of synovitis by distinguishing synovial tissue from effusion. Recently, Dablan et al. [19] reported a reduction in synovitis after GAE using CE-MRI with the semi-quantitative grading system developed by Guermazi et al. [20].

US is a cost-effective and accessible method for assessing biomarkers of synovitis, including effusion, synovial hypertrophy, and Doppler activity [21]. To our knowledge, no studies have yet evaluated the use of US in conjunction with GAE.

As GAE targets vessels, it is crucial to assess the risk of ischemic complications. Three studies reported isolated asymptomatic cases of osteonecrosis-like MRI findings post-GAE [22,23,24], while other studies did not find ischemic lesions on follow-up MRI [13,18,25,26,27,28,29].

This study expands our previous prospective research on GAE patients with mild-to-moderate knee OA, which demonstrated significant improvements in pain relief, functional capacity, quality of life, and physical performance [30]. Our objective is to evaluate changes in synovitis after GAE using MRI and US, while screening for post-procedural ischemic lesions.

## 2. Materials and Methods

This study, conducted at a single center with a prospective, single-arm design, builds upon previous research evaluating patient outcomes after GAE in knee OA patients [30]. Ethical approval was obtained from the local ethical committee (Identifier: H-20081451), and the study was registered on ClinicalTrials.gov (Identifier: NCT05360329). This section provides a brief overview of the study design, including the participant criteria and treatment procedures, as previously described. Additionally, we will outline the US and MRI protocols used in this study, which were not covered in the original publication.

### 2.1. Participants

This study included 17 individuals diagnosed with mild-to-moderate knee OA, as determined by the Kellgren–Lawrence grading system (grades 1–3) [31]. The inclusion criterion was moderate-to-severe knee pain (VAS > 50 mm) during walking, including walking on stairs, despite at least three months of physiotherapy. For participants experiencing bilateral knee pain, the more symptomatic knee was selected for GAE. Key exclusion criteria included a BMI > 35 kg/m^2^, recent intra-articular injections or surgery, and other medical conditions that could interfere with the procedure or imaging.

For patients who met these criteria, baseline imaging assessments, including US and MRI, were performed two days prior to GAE. Follow-up imaging was conducted at one and six months post-procedure.

### 2.2. Genicular Artery Embolization Procedure

An experienced interventional radiologist carried out all GAE interventions. Under local anesthesia, arterial access was obtained via the femoral artery in the groin using a 4-French catheter. Digital subtraction angiography was used to map the vascular anatomy and identify pathological neovessels, which are characterized by a blush-like appearance. These neovessels were selectively catheterized using a 1.7-French microcatheter and guidewire. Embolization was performed using 100–300 µm Embosphere^®^ Microspheres (Merit Medical, South Jordan, UT, USA) diluted in 20 mL of iodinated contrast. The particles were injected gradually until the pathological vessels were occluded. To prevent unintended skin embolization, ice packs were firmly positioned around the knee during the procedure, and a cone-beam CT was used to ensure accuracy. The procedure was deemed technically successful if at least one pathological area of neovessels was embolized. Hemostasis was achieved via manual compression, and participants were monitored for four hours post-procedure before being discharged on the same day. A comprehensive explanation of the GAE technique, including patient selection and follow-up protocols, has been detailed in our previous publication, which this study expands upon [30].

### 2.3. Ultrasound

We used a LOGIQ^TM^ E10 Series US scanner with a ML6-15-D Wideband Matrix Linear Array Probe (GE Healthcare, Chicago, Illinois, USA). All scans were performed in a standardized manner, following the EULAR-scanning guidelines [32]. Synovial hypertrophy, Doppler and effusion were scored separately applying the EULAR-OMERACT scoring system [21]. To ensure accurate and comparable Doppler measurements, Doppler settings were fixed as follows: Color-Doppler; frequency: 8.2 MHz; gain: 23.0; pulse repetition frequency: 0.4 kHz; wall filter: 45; sample volume/placement: 3/16.

**Figure 1:**
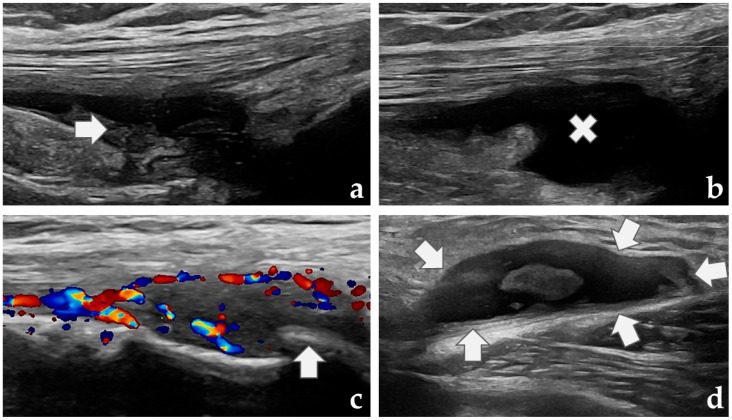
Ultrasound biomarkers of synovitis: (**a**) suprapatellar recess with severe synovial hypertrophy (arrow); (**b**) suprapatellar recess with major effusion (cross); (**c**) lateral parapatellar recess with severe Doppler activity (color) and a moderate osteophyte (arrow); (**d**) Baker’s cyst (arrows) with synovial hypertrophy.

The suprapatellar, medial, and lateral recesses were scanned longitudinally with the patient in a supine position. The knee was flexed to 30 degrees for the suprapatellar recess and naturally extended for the medial and lateral recesses. Synovial hypertrophy and Doppler activity were scored separately from 0 (none) to 3 (severe) in each of the three recesses. Effusion was scored from 0 to 2 (none, minor, major) for the joint overall. Osteophytes at the medial and lateral femorotibial compartments (supine position) and Baker’s cysts at the posteromedial aspect (prone position) were assessed and noted as present or absent. Both the treated and untreated knee were scanned. Examples of the US assessments are shown in Figure 1.

All scans were performed and initially scored by a trained physician (L.H.), with systematic photo and video documentation of all scored areas. These recordings were subsequently reviewed by a recognized expert in musculoskeletal US (L.T.) blinded to the treatment status of the patient. In cases of disagreement, a consensus score was obtained.

### 2.4. MRI

MRI of the target knee was performed using a 3T MAGNETOM Vida scanner equipped with a “Tx/Rx Knee 18” coil (Siemens Healthineers, Erlangen, Germany). The field of view for all sequences was 160 × 160 mm, covering from the suprapatellar recess, proximally, to the patellar tendon insertion, distally. The MRI protocol included coronal T1-weighted turbo spin echo (TSE), axial and sagittal proton density-weighted (PDw) fat-suppressed (FS) TSE, and coronal short tau inversion recovery (STIR) sequences. Additionally, a sagittal CE 3D T1-weighted volumetric interpolated breath-hold examination (VIBE) sequence was performed and reconstructed into axial and coronal planes. Gadolinium (Gadovist, Bayer Healthcare, Berlin, Germany) was used as the contrast agent at a dose of 0.2 mL (0.1 mmol) per kg of body weight. Detailed sequence parameters are provided in the Supplementary Data (S1). The analyses were performed using syngo.via, software version B70hf01 (Siemens Healthineers, Erlangen, Germany).

#### 2.4.1. Non-CE-MRI

We used the axial and sagittal PDw FS TSE images to assess effusion-synovitis according to the MOAKS (MRI Osteoarthritis Knee Score) system [33]. Effusion-synovitis was graded as follows: grade 0 (physiological volume); grade 1 (small—fluid continuous in the retropatellar space); grade 2 (medium—slight convexity of the suprapatellar bursa); and grade 3 (large—evidence of capsular distention). It is important to note that this scoring system is based on a combined evaluation of effusion and thickened synovium, as these two components cannot be reliably distinguished on non-CE-MRI [33].

#### 2.4.2. CE-MRI

**Figure 2:**
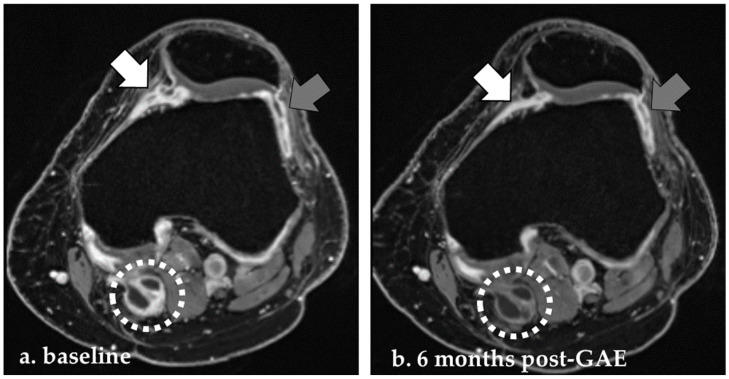
CE-MRI at baseline and 6 months post-GAE. On axial CE-MRI, the synovial thickness of the medial (white arrow) and lateral parapatellar (grey arrow) areas, as well as a possible Baker’s cyst (dotted circle), were included in the whole-joint synovitis score along with areas scored at sagittal images. This patient, treated on the medial side of the left knee, showed a reduction in both local and whole-joint synovitis scores 6 months post-GAE (**b**) compared to baseline (**a**).

We used sagittal and reconstructed axial contrast-enhanced 3D T1-weighted VIBE images to semi-quantitatively assess synovial thickness at 11 anatomical sites within the knee joint, following the method proposed by Guermazi et al. [20]. Synovial thickness at each site was graded as grade 0 (<2 mm), grade 1 (2–4 mm), and grade 2 (>4 mm). The whole-knee synovitis score was calculated by summing the scores from all 11 sites. Additionally, a local synovitis score was determined by including only the treated areas, specifically the parapatellar and parameniscal sites on the treated side of the knee. For participants who were treated on both the medial and lateral sides, all four sites were included in the local synovitis score. Figure 2 shows an example of the axial measurements on a participant at baseline and 6 months post-GAE.

#### 2.4.3. Ischemic Lesions

Coronal T1w TSE and STIR images were examined for signs of newly developed ischemic lesions at both the 1-month and 6-month follow-up scans, with the baseline scan serving as the reference point.

### 2.5. Statistical Analysis

The power calculation for this study was based on the primary endpoint, the Visual Analogue Scale (VAS) at 6 months, as reported in the original publication [30]. Therefore, this study may be underpowered for detecting differences in imaging outcomes. Non-parametric statistical methods were applied. Continuous variables were expressed as medians, while categorical variables were reported as counts. Differences between baseline and follow-up variables at 1 and 6 months were assessed using Friedman’s ANOVA [34]. Post hoc Wilcoxon signed-rank tests were conducted for pairwise comparisons if significant differences were detected [35]. To assess the correlation between US and MRI, effusion and local synovial hypertrophy scores were categorized as “improved”, “stable”, or “worsened” due to non-comparable ordinal scales, and the Cohen’s kappa statistic was applied [36]. A *p*-value of <0.05 was considered statistically significant. All statistical analyses and graphical illustrations were performed using Microsoft Excel (version 2406) and GraphPad Prism (version 10).

## 3. Results

A total of 17 patients were successfully treated and followed up for six months after GAE. Fifteen participants only received GAE on the medial side of the knee, while the remaining two were treated on both the medial and lateral sides. Imaging was performed two days before, as well as one and six months after GAE, with no participants lost to follow-up. Baseline characteristics are shown in Table 1, with some variables repeated from the original publication [30].

### 3.1. Ultrasound

**Figure 3:**
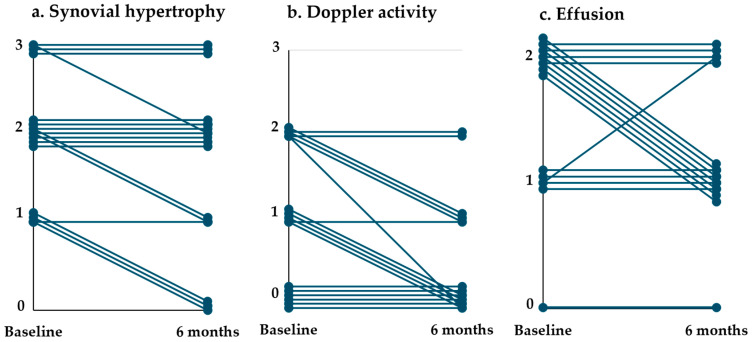
Ultrasound biomarkers of synovitis before and after GAE in the treated areas. Each line represents one participant. *n* = 17. (**a**,**b**): The ultrasound score in the parapatellar recess corresponding to the treated area was used. If both the medial and lateral sides were treated, the highest score was recorded. (**c**): Effusion was assessed across all three recesses.

At the six-month follow-up, local synovial hypertrophy in the treated areas improved in 6 participants and remained stable in 11 (Figure 3a). This change was statistically significant (Friedman’s ANOVA: *p =* 0.030; Wilcoxon signed-rank test: 1 month vs. baseline: *p =* 0.625, 6 months vs. baseline: *p =* 0.031). Local Doppler activity improved in eight participants and remained stable in nine (Figure 3b), also showing statistical significance (Friedman’s ANOVA: *p =* 0.014; Wilcoxon signed-rank test: 1 month vs. baseline: *p =* 0.188, 6 months vs. baseline: *p =* 0.008).

For the untreated areas of the treated knee, significant improvements were observed in both synovial hypertrophy (Friedman’s ANOVA: *p =* 0.005; Wilcoxon signed-rank test: 1 month vs. baseline: *p =* 0.250, 6 months vs. baseline: *p =* 0.016), and Doppler activity (Friedman’s ANOVA: *p =* 0.048; Wilcoxon signed-rank test: 1 month vs. baseline: *p =* 0.094, 6 months vs. baseline: *p =* 0.044). Post hoc tests revealed that the significant differences were primarily driven by changes between baseline and 6 months, as no significant differences were observed at the 1-month follow-up. In the treated knee, effusion improved in seven participants, remained stable in nine, and worsened in one (Figure 3c); however, this change was not statistically significant (Friedman’s ANOVA: *p =* 0.105). The results are summarized in Table 2.

No significant changes were detected in effusion (Friedman’s ANOVA: *p =* 0.595), synovial hypertrophy (*p =* 0.558), or Doppler activity (*p =* 0.974) in the untreated knee from baseline to follow-up, as detailed in Supplementary Data (Table S1).

### 3.2. MRI

#### 3.2.1. Non-CE-MRI—Effusion-Synovitis

**Figure 4:**
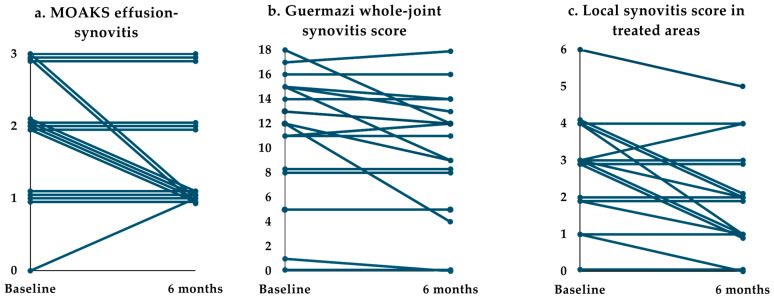
MRI biomarkers of synovitis before and after GAE. Each line represents one participant. *n* = 17. (**a**) MOAKS (MRI Osteoarthritis Knee Score; [33]) from grade 0 to 3, best to worst. (**b**) Guermazi [20] whole-joint synovitis score from 0 to 22, best to worst. (**c**) The local synovitis score is a derived Guermazi score only including treated areas (parameniscal and parapatellar) and ranging from 0 to 8 (0 to 4 for participants only treated at one site of the knee), best to worst.

Six months post-GAE, MOAKS effusion-synovitis [33] had decreased in six, increased in one, and remained stable in ten participants compared to the baseline as illustrated in Table 3 and Figure 4a. However, this change was not statistically significant (Friedman’s ANOVA: *p* = 0.081).

#### 3.2.2. CE-MRI—Synovitis

At the end follow-up, the Guermazi [20] whole-joint synovitis score had decreased in eight, increased in two, and remained stable in seven participants (Table 3, Figure 4b). This change was statistically significant (Friedman’s ANOVA: *p* = 0.045; Wilcoxon signed-rank test 1 month vs. baseline: *p* = 0.027, 6 months vs. baseline: *p* = 0.033).

The local synovitis score had decreased in nine, increased in one, and remained stable in seven participants (Table 3, Figure 4c). This change was statistically significant (Friedman’s ANOVA: *p =* 0.004; Wilcoxon signed-rank test 1 month vs. baseline: *p =* 0.039, 6 months vs. baseline: *p =* 0.012).

#### 3.2.3. Agreement Between US and MRI Scores

Participants were categorized as improved, stable, or worsened based on changes in the US-assessed local synovial hypertrophy score from baseline to 6 months, and similarly for the local MRI synovitis score, which was also based on synovial thickness. A Cohen’s kappa score of 0.57 indicated a moderate agreement between the two modalities. The MRI-assessed effusion-synovitis and US-assessed effusion were compared using the same method, yielding a similar Cohen’s kappa score of 0.56. Detailed heat maps for both comparisons are provided in Supplementary Data (Figures S1 and S2).

#### 3.2.4. Ischemic Lesions

**Figure 5:**
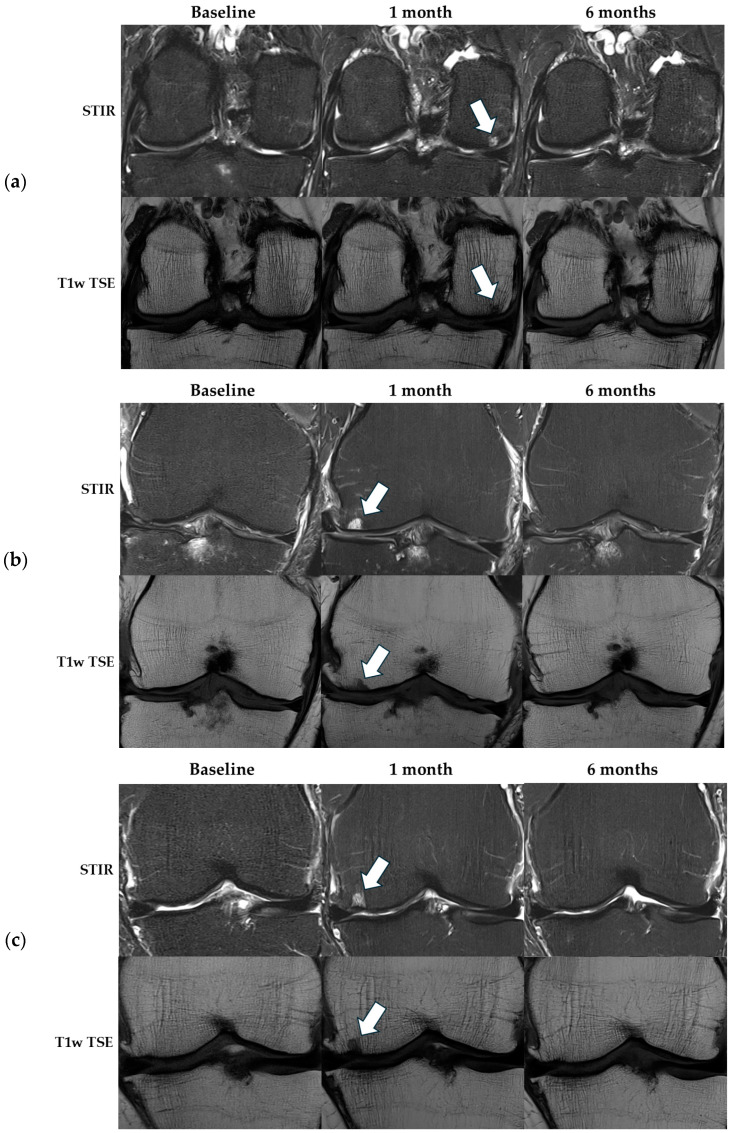
Ischemic-like lesions on MRI. Three cases of nonspecific ischemic-like lesions one month after GAE (white arrows), all completely resolved by six months. The three participants were embolized in the following areas: (**a**) medial side of the right knee, (**b**) medial and lateral side of the right knee, and (**c**) medial side of the left knee. In all three cases, the lesions corresponded to the treated areas.

At the 1-month follow-up MRI, three participants exhibited newly developed, atypical subchondral lesions with sharply defined borders, displaying low signal intensity on T1-weighted images, and high signal intensity on STIR sequences. However, by the 6-month follow-up, these lesions had completely resolved with no sequelae (Figure 5). Three radiologists, with 22, 9, and 5 years of musculoskeletal experience, were uncertain about whether these areas indicated temporary localized ischemia without necrosis, or normal, fluctuating subchondral reactions due to underlying OA.

## 4. Discussion

This prospective study demonstrated a statistically significant reduction in synovitis after GAE, as measured by MRI and US biomarkers, observed not only in the embolized areas but also throughout the treated knee. Although effusion decreased, the change was not statistically significant, possibly due to the small sample size and potential type II errors. US examination of the untreated knee showed no changes. MRI at one month showed three cases of osteonecrosis-like areas, all of which were resolved by six months without any lasting ischemic complications.

Studies by Okuno et al. [18] and Little et al. [13] investigated changes in effusion/synovitis after GAE with the semi-quantitative scoring system WORMS [17], finding significant improvements two years and one year after GAE, respectively. However, non-CE-MRI, in contrast to CE-MRI, does not allow for differentiation between effusion and synovium. Effusion can be influenced by day-to-day activity, while synovial thickening is a better indicator of chronic inflammation. Dablan et al. [19] used a similar approach to ours, using the CE-MRI scoring system by Guermazi et al. [20], based on synovial thickness, and found a significant reduction in both local and whole-joint synovitis. CE-MRI, however, can be challenging in clinical settings due to the cost and patient contraindications.

To our knowledge, no other studies have utilized US in relation to GAE. US, like CE-MRI, can differentiate between effusion and synovium. Additionally, Doppler US can serve as a biomarker for the blood flow in the tissue, which is of great interest in this treatment approach that specifically targets and occludes blood vessels. US is a cost-effective and widely accessible method with significant potential for both screening GAE candidates and conducting follow-up assessments.

At the 1-month follow-up MRI, three participants showed atypical osteonecrosis-like subchondral lesions in the areas corresponding to the embolization. Five similar asymptomatic cases were reported in three previous studies at 1- and 3-month follow-up scans [22,23,24]. Other studies have specifically reported that no ischemic lesions were found on MRI. In our study, all participants had a second follow-up MRI at 6 months and, at this stage, all these lesions had completely resolved with no sequelae. Based on these findings, three consulting specialists suggested that the observed areas might represent temporary localized ischemia or incidental findings of normal fluctuating subchondral reactions related to the underlying OA condition. Given that GAE involves the targeted pruning of blood vessels, it is crucial to thoroughly investigate any potential ischemic complications related to the procedure. Our results highlight the importance of long-term follow-up and careful consideration of the patients’ underlying disease when interpreting imaging parameters, distinguishing between fluctuations in their baseline condition and the possible effects of the treatment.

Our study has several limitations, including a small sample size, the lack of a control group, and limited follow-up duration. The sample size was based on power calculations for the primary VAS outcome in the original study [30], which may be insufficient for robust imaging analyses, increasing the risk of type II errors. When comparing US and MRI findings, the use of validated scoring systems with different ordinal scales prevented direct comparisons. In small datasets, Cohen’s kappa can produce an erroneously low value, as even a few outliers or disagreements can disproportionately affect the overall measure of agreement. The untreated knee could not serve as a control due to variability in the participants’ conditions; 11 participants had bilateral knee pain while 6 did not, and the sample size hindered meaningful subgroup analyses. Despite these limitations, our findings provide preliminary evidence that merits further investigation in larger studies with longer follow-up periods to establish more robust conclusions.

Future GAE studies should investigate whether pre-procedural imaging can predict the presence of an angiographic hyperemic blush. This requires standardized angiographic procedures because variations in microcatheter placement and contrast injection techniques affect the appearance of the blush. Additionally, research should explore whether pre-procedural imaging biomarkers of synovitis can predict treatment response, as suggested by prior studies [19,25,37,38], and whether post-procedural imaging correlates with patient-reported outcomes. These strategies could enhance patient selection, safety, satisfaction, and cost-effectiveness. Furthermore, future studies should assess benefits beyond pain relief, such as slowing joint destruction by reducing inflammation, ideally through larger controlled trials with extended follow-up periods. MRI studies should also employ appropriate sequences to evaluate and report ischemic complications.

## 5. Conclusions

This study provides preliminary evidence suggesting that GAE may effectively reduce synovitis in patients with mild-to-moderate knee OA, as demonstrated by US and MRI assessments. Statistically significant improvements were observed in synovial hypertrophy and Doppler activity, while reductions in effusion were noted but did not reach statistical significance. Importantly, no permanent ischemic complications were detected, as the three transient osteonecrosis-like areas observed at the one-month follow-up resolved completely by six months. Further research with larger cohorts and a longer follow-up is needed to confirm the safety and efficacy of GAE for managing knee OA.

## Figures and Tables

**Table 1:** Baseline characteristics.

*n* = 17		Treated Knee	Contralateral Knee
Women/Men, *n* (%)	9 (53%)/8 (47%)		
Age, years, median (range)	56 (43–71)		
BMI, kg/m^2^, median (range)	27 (20–35)		
Bilateral knee pain, *n* (%)	11 (65%)		
Embolized knee, right/left, *n* (%)		10 (59%)/7 (41%)	
Localization of treatment			
*Only medial, n (%)*		15 (88%)	
*Medial and lateral, n (%)*		2 (12%)	
X-ray KL grade			
*1, n (%)*		2 (12%)	
*2, n (%)*		7 (41%)	
*3, n (%)*		8 (47%)	
Baker’s cysts, *n* (%) *		9 (53% **)	5 (29%)
Osteophytes, *n* (%) *		15 (88%)	14 (82%)
Pathological effusion, *n* (%) *		11 (65%)	6 (35%)

Baseline data presented in this table include variables previously reported in Hindsø et al., 2024 [30]. * Ultrasound diagnosis. ** One participant had earlier Baker’s cyst removal surgery and was not included. BMI, body mass index; KL, Kellgren-Lawrence.

**Table 2:** Ultrasound biomarkers of synovitis before and after GAE.

**a. Embolized Area in Treated Knee**				
***n* = 17**	**Baseline**	**1 Month**	**6 Months**	**Friedman’s ANOVA**
**Effusion**				X^2^ (2) = 4.5, *p =* 0.105
Grade 0, *n*	1	0	1	
Grade 1, *n*	5	10	11	
Grade 2, *n*	11	7	5	
**Synovial Hypertrophy**				X^2^ (2) = 7.0, *p =* 0.030
Grade 0, *n*	0	0	3	
Grade 1, *n*	4	6	3	
Grade 2, *n*	9	7	8	
Grade 3, *n*	4	4	3	
**Doppler activity**				X^2^ (2) = 8.6, *p =* 0.014
Grade 0, *n*	6	10	11	
Grade 1, *n*	5	3	4	
Grade 2, *n*	6	3	2	
Grade 3, *n*	0	1	0	
Each number in the table represents the count of participants for the respective severity grade. Effusion was assessed across all three recesses. For synovial hypertrophy and Doppler activity, the medial parapatellar recess score is shown for the 15 participants treated only on the medial side of the knee. For the two participants treated on both sides, the highest severity score between the medial and lateral sides is reported.				
**b. Non-Embolized Areas of the Treated Knee**				
***n* = 17**	**Baseline**	**1 Month**	**6 Months**	**Friedman’s ANOVA**
**Synovial Hypertrophy**				X^2^ (2) =10.6, *p =* 0.005
Grade 0, *n*	0	0	1	
Grade 1, *n*	2	3	5	
Grade 2, *n*	8	10	7	
Grade 3, *n*	7	4	4	
**Doppler activity**				X^2^ (2) = 6.0, *p =* 0.048
Grade 0, *n*	4	8	10	
Grade 1, *n*	7	5	4	
Grade 2, *n*	5	4	3	
Grade 3, *n*	1	0	0	
Each number in the table represents the count of participants for the respective severity grade. For the 15 participants treated only on the medial side of the knee, the highest scores recorded in the lateral parapatellar and the suprapatellar recesses were used. For the two participants treated on both the medial and lateral sides, the score from the suprapatellar recess was used.				

**Table 3:** Non-CE- and CE-MRI before and after GAE.

	Baseline	1 Month	6 Months	Friedman’s ANOVA
**Non-CE-MRI—MOAKS** **Effusion-synovitis**				X^2^ (2) = 5.0, *p =* 0.081
Grade 0, *n*	1	1	0	
Grade 1, *n*	4	6	11	
Grade 2, *n*	7	7	3	
Grade 3, *n*	5	3	3	
**CE-MRI—Guermazi**				
Whole-joint synovitis, median [IQR]	12 [8;15]	11 [8;14]	11 [6;13]	X^2^ (2) = 6.2, *p =* 0.045
Local synovitis, median [IQR]	3 [2;4]	2 [1.5;3]	2 [1;3]	X^2^ (2) = 11.1, *p =* 0.004

MOAKS (MRI Osteoarthritis Knee Score; [33]) from grade 0 to 3, best to worst. Guermazi [20] whole-joint synovitis score ranging from 0 to 22, best to worst. The local synovitis score is a derived Guermazi score only including treated areas (parameniscal and parapatellar) and ranging from 0 to 8 (0 to 4 for participants only treated at one site of the knee), best to worst.

## Data Availability

The datasets used and analyzed during the current study are available from the corresponding author on reasonable request.

## Supplementary Materials

The following supporting information can be downloaded at: https://www.mdpi.com/article/10.3390/diagnostics14222564/s1, S1: MRI protocol; Table S1: Ultrasound biomarkers of synovitis before and after GAE in the non-treated knee; Figure S1: changes in MRI vs. ultrasound-assessed synovial hypertrophy; Figure S2: changes in MRI-assessed effusion-synovitis vs. ultrasound-assessed effusion.

## References

[1] Weng Q., Chen Q., Jiang T., Zhang Y., Zhang W., Doherty M., Xie J., Liu K., Li J., Yang T. (2024). Global Burden of Early-Onset Osteoarthritis, 1990–2019: Results from the Global Burden of Disease Study 2019. Ann. Rheum. Dis..

[2] WPHO Scientific Group on the Burden of Musculoskeletal Conditions at the Start of the New Millennium (2003). The Burden of Musculoskeletal Conditions at the Start of the New Millennium.

[3] Hunter D.J., Bierma-Zeinstra S. (2019). Osteoarthritis. Lancet.

[4] Leifer V.P., Katz J.N., Losina E. (2022). The Burden of OA-Health Services and Economics. Osteoarthr. Cartil..

[5] Sharma L. (2021). Osteoarthritis of the Knee. N. Engl. J. Med..

[6] Yue L., Berman J. (2022). What Is Osteoarthritis?. JAMA.

[7] Krakowski P., Rejniak A., Sobczyk J., Karpiński R. (2024). Cartilage Integrity: A Review of Mechanical and Frictional Properties and Repair Approaches in Osteoarthritis. Healthcare.

[8] Bonnet C.S., Walsh D.A. (2005). Osteoarthritis, Angiogenesis and Inflammation. Rheumatology.

[9] Hayashi D., Roemer F.W., Guermazi A. (2019). Imaging of Osteoarthritis by Conventional Radiography, MR Imaging, PET–Computed Tomography, and PET–MR Imaging. PET Clin..

[10] Mathiessen A., Cimmino M.A., Hammer H.B., Haugen I.K., Iagnocco A., Conaghan P.G. (2016). Imaging of Osteoarthritis (OA): What Is New?. Best Pract. Res. Clin. Rheumatol..

[11] Epelboym Y., Mandell J.C., Collins J.E., Burch E., Shiang T., Killoran T., Macfarlane L., Guermazi A. (2023). Genicular Artery Embolization as a Treatment for Osteoarthritis Related Knee Pain: A Systematic Review and Meta-Analysis. Cardiovasc. Intervent. Radiol..

[12] Hindsø L., Riis R.G.C., Hölmich P., Petersen M.M., Nielsen M.B., Lönn L., Taudorf M. (2021). Current Status of Trans-Arterial Embolization in Pain Management of Musculoskeletal Inflammatory Conditions—An Evidence-Based Review. Cardiovasc. Intervent. Radiol..

[13] Little M.W., O’Grady A., Briggs J., Gibson M., Speirs A., Al-Rekabi A., Yoong P., Ariyanayagam T., Davies N., Tayton E. (2024). Genicular Artery Embolisation in Patients with Osteoarthritis of the Knee (GENESIS) Using Permanent Microspheres: Long-Term Results. Cardiovasc. Intervent. Radiol..

[14] Okuno Y., Korchi A.M., Shinjo T., Kato S. (2015). Transcatheter Arterial Embolization as a Treatment for Medial Knee Pain in Patients with Mild to Moderate Osteoarthritis. Cardiovasc. Intervent. Radiol..

[15] Epelboym Y., Lee L., Okuno Y., Korchi A. (2023). Genicular Artery Embolization as a Treatment for Refractory Osteoarthritis Related Knee Pain. Skelet. Radiol..

[16] Casadaban L.C., Mandell J.C., Epelboym Y. (2021). Genicular Artery Embolization for Osteoarthritis Related Knee Pain: A Systematic Review and Qualitative Analysis of Clinical Outcomes. Cardiovasc. Intervent. Radiol..

[17] Peterfy C.G., Guermazi A., Zaim S., Tirman P.F.J., Miaux Y., White D., Kothari M., Lu Y., Fye K., Zhao S. (2004). Whole-Organ Magnetic Resonance Imaging Score (WORMS) of the Knee in Osteoarthritis. Osteoarthr. Cartil..

[18] Okuno Y., Korchi A.M., Shinjo T., Kato S., Kaneko T. (2017). Midterm Clinical Outcomes and MR Imaging Changes after Transcatheter Arterial Embolization as a Treatment for Mild to Moderate Radiographic Knee Osteoarthritis Resistant to Conservative Treatment. J. Vasc. Interv. Radiol..

[19] Dablan A., Erdim Ç., Güzelbey T., Cingöz M., Arslan M.F., Mutlu İ.N., Kılıçkesmez Ö. (2024). Effectiveness of Genicular Artery Embolization for Reducing Synovitis as Assessed by Contrast-Enhanced MR Imaging in Knee Osteoarthritis: A Pilot Study. J. Vasc. Interv. Radiol. JVIR.

[20] Guermazi A., Roemer F.W., Hayashi D., Crema M.D., Niu J., Zhang Y., Marra M.D., Katur A., Lynch J.A., El-Khoury G.Y. (2011). Assessment of Synovitis with Contrast-Enhanced MRI Using a Whole-Joint Semiquantitative Scoring System in People with, or at High Risk of, Knee Osteoarthritis: The MOST Study. Ann. Rheum. Dis..

[21] Terslev L., Naredo E., Aegerter P., Wakefield R.J., Backhaus M., Balint P., Bruyn G.A.W., Iagnocco A., Jousse-Joulin S., Schmidt W.A. (2017). Scoring Ultrasound Synovitis in Rheumatoid Arthritis: A EULAR-OMERACT Ultrasound Taskforce-Part 2: Reliability and Application to Multiple Joints of a Standardised Consensus-Based Scoring System. RMD Open.

[22] Padia S.A., Genshaft S., Blumstein G., Plotnik A., Kim G.H.J., Gilbert S.J., Lauko K., Stavrakis A.I. (2021). Genicular Artery Embolization for the Treatment of Symptomatic Knee Osteoarthritis. JBJS Open Access.

[23] Taslakian B., Swilling D., Attur M., Alaia E.F., Kijowski R., Samuels J., Macaulay W., Ramos D., Liu S., Morris E.M. (2023). Genicular Artery Embolization for Treatment of Knee Osteoarthritis: Interim Analysis of a Prospective Pilot Trial Including Effect on Serum Osteoarthritis-Associated Biomarkers. J. Vasc. Interv. Radiol..

[24] Bagla S., Piechowiak R., Hartman T., Orlando J., Del Gaizo D., Isaacson A. (2020). Genicular Artery Embolization for the Treatment of Knee Pain Secondary to Osteoarthritis. J. Vasc. Interv. Radiol..

[25] Landers S., Hely R., Page R., Maister N., Hely A., Harrison B., Gill S. (2020). Genicular Artery Embolization to Improve Pain and Function in Early-Stage Knee Osteoarthritis-24-Month Pilot Study Results. J. Vasc. Interv. Radiol..

[26] Bagla S., Piechowiak R., Sajan A., Orlando J., Hartman T., Isaacson A. (2022). Multicenter Randomized Sham Controlled Study of Genicular Artery Embolization for Knee Pain Secondary to Osteoarthritis. J. Vasc. Interv. Radiol. JVIR.

[27] Landers S., Hely R., Hely A., Harrison B., Page R.S., Maister N., Gwini S.M., Gill S.D. (2023). Genicular Artery Embolization for Early-Stage Knee Osteoarthritis: Results from a Triple-Blind Single-Centre Randomized Controlled Trial. Bone Jt. Open.

[28] Sun C., Chen Y., Gao Z., Wu L., Lu R., Zhao C., Yang H., Chen Y. (2024). Genicular Artery Embolization for the Treatment of Knee Pain Secondary to Mild to Severe Knee Osteoarthritis: One Year Clinical Outcomes. Eur. J. Radiol..

[29] Gill S.D., Hely R., Hely A., Harrison B., Page R.S., Landers S. (2023). Outcomes after Genicular Artery Embolization Vary According to the Radiographic Severity of Osteoarthritis: Results from a Prospective Single-Center Study. J. Vasc. Interv. Radiol. JVIR.

[30] Hindsø L., Hölmich P., Petersen M.M., Nielsen M.B., Heerwagen S., Taudorf M., Lönn L. (2024). Transarterial Embolization of Geniculate Arteries Reduces Pain and Improves Physical Function in Knee Osteoarthritis-A Prospective Cohort Study. Diagnostics.

[31] Kellgren J.H., Lawrence J.S. (1957). Radiological Assessment of Osteo-Arthrosis. Ann. Rheum. Dis..

[32] Möller I., Janta I., Backhaus M., Ohrndorf S., Bong D.A., Martinoli C., Filippucci E., Sconfienza L.M., Terslev L., Damjanov N. (2017). The 2017 EULAR Standardised Procedures for Ultrasound Imaging in Rheumatology. Ann. Rheum. Dis..

[33] Hunter D.J., Guermazi A., Lo G.H., Grainger A.J., Conaghan P.G., Boudreau R.M., Roemer F.W. (2011). Evolution of Semi-Quantitative Whole Joint Assessment of Knee OA: MOAKS (MRI Osteoarthritis Knee Score). Osteoarthr. Cartil..

[34] Friedman M. (1937). The Use of Ranks to Avoid the Assumption of Normality Implicit in the Analysis of Variance. J. Am. Stat. Assoc..

[35] Wilcoxon F. (1945). Individual Comparisons by Ranking Methods. Biom. Bull..

[36] McHugh M.L. (2012). Interrater Reliability: The Kappa Statistic. Biochem. Medica.

[37] Choi J.W., Ro D.H., Chae H.D., Kim D.H., Lee M., Hur S., Kim H.-C., Jae H.J., Chung J.W. (2020). The Value of Preprocedural MR Imaging in Genicular Artery Embolization for Patients with Osteoarthritic Knee Pain. J. Vasc. Interv. Radiol..

[38] van Zadelhoff T.A., Okuno Y., Bos P.K., Bierma-Zeinstra S.M.A., Krestin G.P., Moelker A., Oei E.H.G. (2021). Association between Baseline Osteoarthritic Features on MR Imaging and Clinical Outcome after Genicular Artery Embolization for Knee Osteoarthritis. J. Vasc. Interv. Radiol..

