# Supplementary figures and images for: Reduction in Synovitis Following Genicular Artery Embolization in Knee Osteoarthritis: A Prospective Ultrasound and MRI Study
Source: Diagnostics (Basel). 2024 Nov 15;14(22):2564. doi: 10.3390/diagnostics14222564 (PMC11592601; doi:10.3390/diagnostics14222564)

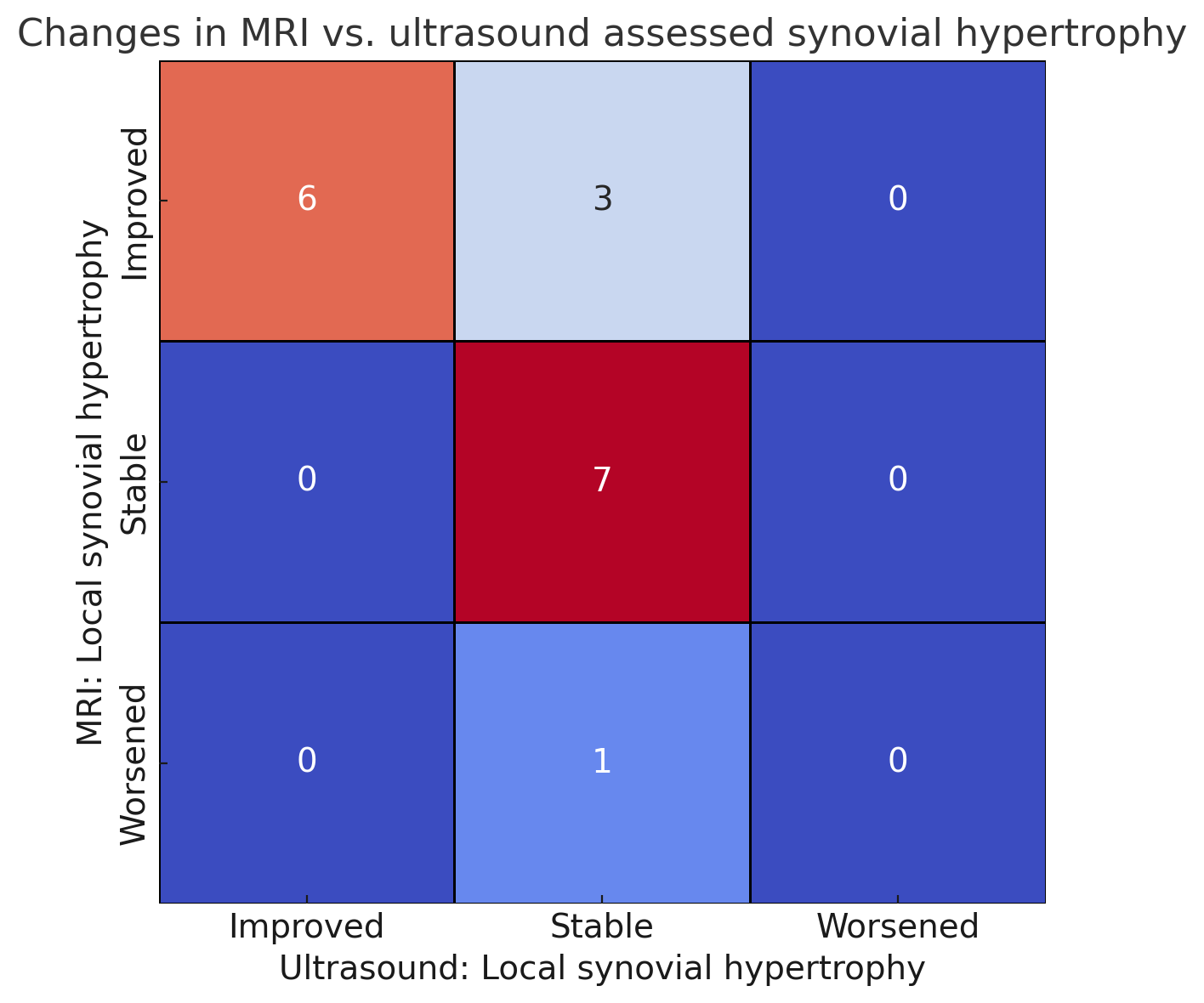

Supplement: Supplementary file 1 [file diagnostics-14-02564-s001.zip › Supplementary/Figure S1.png]

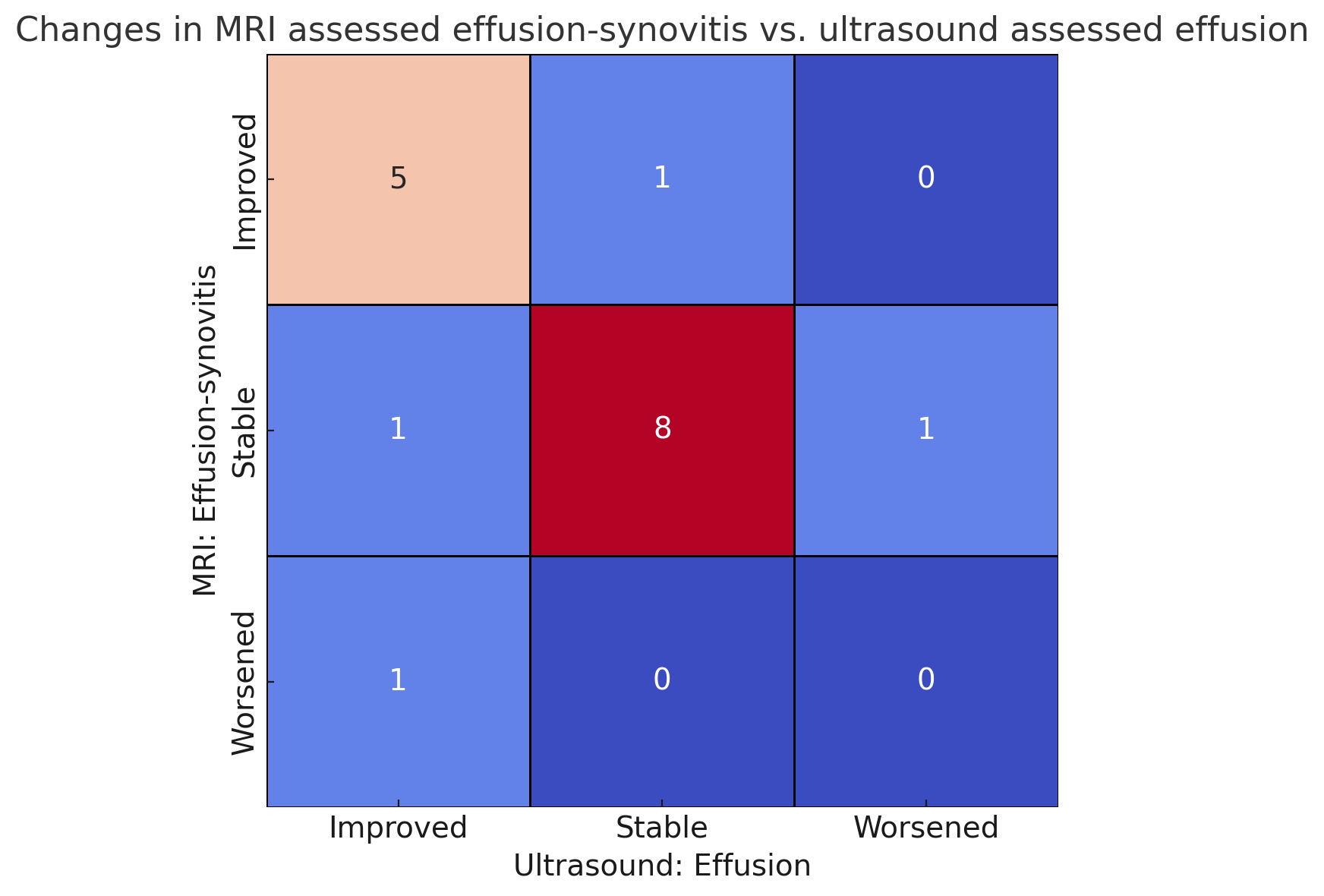

Supplement: Supplementary file 1 [file diagnostics-14-02564-s001.zip › Supplementary/Figure S2.png]

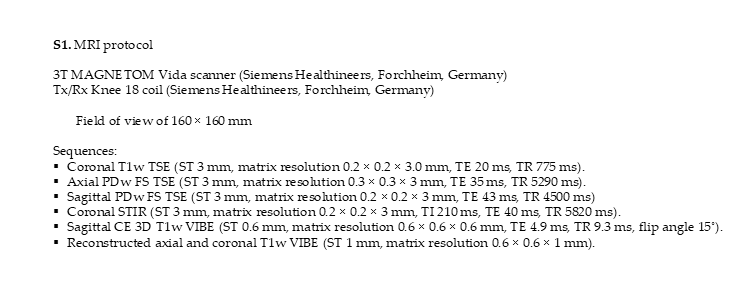

Supplement: Supplementary file 1 [file diagnostics-14-02564-s001.zip › Supplementary/S1.png]

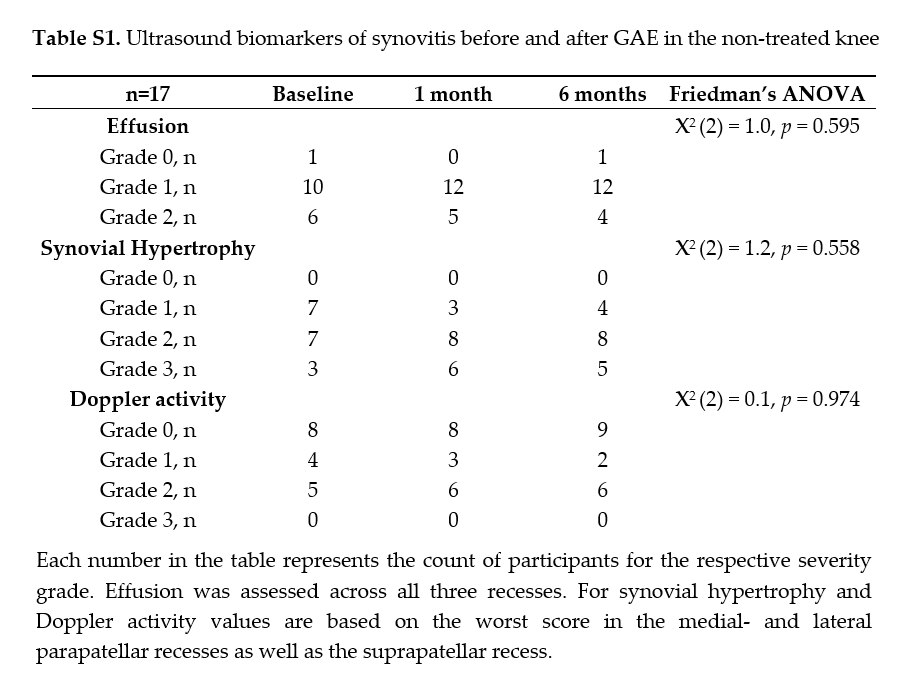

Supplement: Supplementary file 1 [file diagnostics-14-02564-s001.zip › Supplementary/Table S1.png]
